# Phylogeographic analysis reveals extensive genetic variation of native grass *Elymus nutans* (Poaceae) on the Qinghai-Tibetan plateau

**DOI:** 10.3389/fpls.2024.1349641

**Published:** 2024-03-11

**Authors:** Jin Li, Xinda Li, Changbing Zhang, Qingping Zhou, Shiyong Chen

**Affiliations:** ^1^ Sichuan Zoige Alpine Wetland Ecosystem National Observation and Research Station, Southwest Minzu University, Chengdu, China; ^2^ College of Animal and Veterinary Sciences, Southwest Minzu University, Chengdu, China; ^3^ Institute of Grass Plants, Sichuan Academy of Grassland Science, Chengdu, China

**Keywords:** *Elymus nutans*, Qinghai-Tibet plateau, phylogeography, chloroplast DNA, EST-SSR

## Abstract

**Introduction:**

*Elymus nutans* holds ecological and pastoral significance due to its adaptability and nutritional value, the Qinghai-Tibet Plateau (QTP) is a key hub for its genetic diversity. To conserve and harness its genetic resources in highland ecosystems, a thorough assessment is vital. However, a comprehensive phylogeographic exploration of *E. nutans* is lacking. The objective of this study was to unravel the genetic diversity, adaptation, and phylogenetics of *E. nutans* populations.

**Methods:**

Encompassing 361 individuals across 35 populations, the species' genetic landscape and dynamic responses to diverse environments were decoded by using four chloroplast DNA (cpDNA) sequences and nine microsatellite markers derived from the transcriptome.

**Results and discussion:**

This study unveiled a notable degree of genetic diversity in *E. nutans* populations at nuclear (I = 0.46, He = 0.32) and plastid DNA levels (Hd = 0.805, π = 0.67). Analysis via AMOVA highlighted genetic variation predominantly within populations. Despite limited isolation by distance (IBD), the Mekong-Salween Divide (MSD) emerged as a significant factor influencing genetic differentiation and conserving diversity. Furthermore, correlations were established between external environmental factors and effective alleles of three EST-SSRs (EN5, EN57 and EN80), potentially linked to glutathione S-transferases T1 or hypothetical proteins, affecting adaptation. This study deepens the understanding of the intricate relationship between genetic diversity, adaptation, and environmental factors within *E. nutans* populations on the QTP. The findings shed light on the species' evolutionary responses to diverse ecological conditions and contribute to a broader comprehension of plant adaptation mechanisms.

## 1 Introduction

The Qinghai-Tibet Plateau (QTP), known as the “Roof of the World,” holds significant importance in phylogeographic research due to its exceptional biodiversity and ecological significance (Yu et al., 2019). It serves as a center for genetic differentiation, supporting numerous endemic genera and species (Shahzad et al., 2017). The unique biodiversity of the plateau can be attributed to Quaternary climate oscillations and the uplift stages during the Miocene-Pliocene or Miocene-Quaternary periods (Zheng et al., 2021). Furthermore, compared to other regions, the QTP exhibits heightened sensitivity to Quaternary climate change, resulting in notable effects on the geographic and population distribution of plant species and leaving distinct genetic imprints (Du et al., 2020). However, the current rate of global climate change is unprecedented in history, raising concerns about its potential impact on species distribution and diversity (Proft et al., 2021). This risk is particularly alarming for the QTP, which is home to numerous endemic and threatened species highly susceptible to climate variations (Wang et al., 2018; Sun et al., 2021). Ensuring their survival is a matter of utmost concern. In response to rapid climate change, natural populations primarily exhibit adaptation, migration, or extinction (Stojanova et al., 2018). Genetic variation plays a pivotal role in species’ capacity to adapt to dynamic and ever-changing environments, when confronted with climate change, populations with higher genetic diversity possess an enhanced likelihood of survival (Gray et al., 2014). This heightened genetic diversity enables them to better withstand the intense selection pressures imposed by contemporary climate change (Jump et al., 2009).

Previous phylogeographic investigations have effectively compared and assessed the plant populations in the QTP, fostering explorations into the intricate interactions between lineage evolution, environmental heterogeneity, and genetic diversity (Aguiar-Melo et al., 2019; Liu et al., 2018). However, it’s noteworthy that while extensive phylogeographic surveys have been conducted on the flora of the QTP, a considerable portion of these studies has predominantly focused on arboreal and shrub species (Kou et al., 2014), often overlooking herbaceous plants (Xiong et al., 2021). Herbaceous plants, with their exceptional cold tolerance, temperature adaptability, and drought resistance, are widely distributed across the plateau, exhibiting unique adaptability and ecological relevance (Wang and Lu, 2014; Sun et al., 2020). Therefore, understanding the phylogenetic history and genetic status of these Poaceae plants is crucial for a comprehensive understanding of the floral evolution on the Qinghai-Tibet Plateau and for the formulation of effective germplasm conservation strategies (Lei et al., 2022).

The majority of herbaceous plants possess intricate evolutionary histories and large genomes, significantly impeding the application of genome scanning techniques and consequently imposing limitations on phylogeographic research efforts (Zwyrtková et al., 2022). Given the crucial role of grasslands in the QTP, this knowledge gap becomes even more consequential (Liu et al., 2018). In such scenarios, expressed sequence tag simple sequence repeats (EST-SSRs) derived from transcriptome sequencing data have emerged as a reliable alternative (Vu et al., 2020; Li et al., 2023). Typically located in functional gene regions, EST-SSRs offer a plethora of advantages including high polymorphism, broad applicability to closely related species, repeatability, and cost-efficiency (Decroocq et al., 2003). Moreover, maternal chloroplast markers, characterized by their relatively low mutation rates and stable genetic structures, are deemed ideal tools for deciphering population genetic structures and evolutionary histories (Birky et al., 1983). These markers, due to their maternal inheritance, provide researchers with a means to circumvent complex genome structures and diverse genetic backgrounds, thereby facilitating more precise tracing of lineage relationships and genetic drift (Provan et al., 2001), and proving particularly invaluable in resolving phylogenetic admixture and reconstructing population dispersal paths (Petit et al., 1993). Consequently, in phylogeographic and evolutionary biological studies, the amalgamation of EST-SSRs and maternal chloroplast markers offers valuable genetic information for species with limited genomic resources. This genetic information can be effectively utilized across various research domains. For instance, Setsuko et al. (2020) combined 14 EST-SSRs with 3 cpDNA markers (*trn*Q-*rps*16, *mat*K, and *trn*L-*trn*F) to investigate the population diversity and structure of the endemic plant *Pandanus boninensis* in the Ogasawara Islands, Japan, gathering evidence of migration between older and younger islands. Guo et al. (2022) utilized 38 EST-SSRs and 3 cpDNA markers (*ndh*F-*rpl*132, *rps*16-*trn*Q, and *trn*E-*trn*T) to test the refugia hypothesis for the subtropical vine plant *Actinidia eriantha* in Eastern China.

The current investigation delineates the geographic structure and population divergence of *Elymus nutans*, a perennial and allohexaploid (2n = 6x = 42) entity within the Triticeae (Poaceae). As an extensively distributed herbaceous plant, boasts a widespread presence across the QTP and the Himalayas, thriving at elevations ranging from 3000 to 4500 m (Chen et al., 2009). Owing to its prolific yield, nutritional richness, and robustness against a spectrum of abiotic stresses, this species assumes a critical role in animal husbandry and environmental conservation. These attributes position *E. nutans* as an exemplary subject for initiatives in ecological restoration, development of artificial grasslands, and advancement of agricultural and ecological studies in the QTP. However, the habitat of indigenous plants in the QTP, including *E. nutans*, has been threatened by climate change and overgrazing in recent decades (Pradheep et al., 2019). Therefore, understanding the genetic status of *E. nutans* germplasm resources is critical for the conservation and utilization of genetic resources, and maintaining the ecosystem stability. In the present study, the EST-SSR markers were used to assess the genetic diversity of *E. nutans* populations and obtain inter-population gene flow information, and the sequence variations in four chloroplast DNA regions were also applied to analyze the phylogeographic structuring of *E. nutans* populations. This research endeavors to demystify how the genetic variations identified contribute to the species’ capacity to adapt to the diverse environmental conditions prevalent across the QTP. Furthermore, it seeks to determine whether the relationships established between genetic markers and external environmental influences can provide a deeper understanding of the inherent adaptive mechanisms of the species. The findings of this investigation have the potential to enhance our understanding of germplasm resources and inform breeding strategies for this species.

## 2 Methods

### 2.1 Plant sampling and DNA extraction

In the present study, 361 silica-dried leaf samples from 35 populations of *E. nutans* were collected in the southeastern QTP from August to September 2019. In order to reveal the genetic characteristics of this species in response to environmental change, populations sampling in this study was carried out on a longitude gradient. For each population, we sampled 7 to 15 individuals, ensuring that the sampling locations were at least 5 meters apart. The seeds of these specimens were planted at the experimental field of Sichuan Academy of Grassland Science, Hongyuan, China (32°46.61’N, 102°32.63’E). **Supplementary Table 1** provides detailed information regarding the collection sites, geographical coordinates, and elevations for each of the source populations. Genomic DNA extraction was carried out using the DP350 Plant DNA Kit (Tiangen Biotechnology, Beijing, China), following the manufacturer’s protocol. Subsequently, the quality and quantity of the extracted DNA samples were assessed using a NanoDrop-Lite instrument (Thermo Scientific, Waltham, MA, USA) and 1% agarose gel electrophoresis, respectively. Only the DNA samples meeting the required quality criteria were considered for further analysis. Subsequently, the qualified DNA samples were diluted to a concentration of 10 ng/μL for use.

### 2.2 Chloroplast DNA sequencing and EST-SSR genotyping

Four cpDNA fragments (*trn*H-*psb*A, *trn*L-F, *mat*K, and *rbc*L) were amplified and sequenced across all *E. nutans* samples. These fragments were amplified using primers previously reported in the literature, known for showcasing polymorphisms in other *Elymus* species (Xiong et al., 2022). Amplification was conducted in a 30 μL reaction volume, comprising of 30 ng of genomic DNA, 1.5 μL of each primer, and 15 μL of 2× Es Taq MasterMix (CoWin Biosciences, Beijing, China), using a C1000 Touch Thermal Cycler (BIO-RAD, Foster City, CA, USA). The polymerase chain reaction (PCR) conditions throughout the experiment were consistent with those described by Shaw et al (Shaw et al., 2005), and the resulting products were sequenced by Tsingke Biotech (Beijing, China). The genotypes of all 361 samples were determined using nine pairs of EST-SSR primers developed for *E. nutans* in our previous study based on transcriptomic data, and the PCR procedure followed the same protocol as we described previously (Li et al., 2023). The PCR products were subjected to capillary electrophoresis in an ABI 3730xl DNA analyzer (Applied Biosystems, Foster, CA, USA) and analyzed using GeneMarker v. 2.2 software (SoftGenetics, Pennsylvania, USA).

### 2.3 Phylogeographic analysis based on cpDNA sequencing

All cpDNA data were edited and adjusted using DNAMAN to obtain consensus sequences. The sequences were then aligned using the ClustalW algorithm in MEGA 6.0 (Tamura et al., 2013). PhyloSuite v1.2.3 was utilized to concatenate the sequences serially (Zhang et al., 2020). DnaSP v5.0 was employed to calculate haplotype (H), haplotype diversity (*H*d), nucleotide diversity (*π*), and population genetic distance based on the concatenated sequences (Librado and Rozas, 2009). A haplotype network was constructed using the median-joining method in PopART software (Leigh and Bryant, 2015). The haplotype phylogenetic tree was constructed by the maximum likelihood (ML) method in MEGA 6.0 software, *Elymus repens* and *Elymus ciliaris* were chosen as outgroups (Bouckaert et al., 2014). The maximum clade credibility tree and additional summary statistics were visualized using FigTree 1.3.1. This enabled the assessment of potential spatial expansion within the populations, Tajima’s D and Fu’s Fs were calculated using DnaSP version 5.0. Mismatch distribution analysis and neutrality tests were employed (Tajima, 1989; Fu and Li, 1993). Analysis of molecular variance (AMOVA) was conducted using Arlequin v3.5 to estimate genetic variance within and between populations (Excoffier and Lischer, 2010).

### 2.4 Population structure and genetic barriers analysis based on microsatellite data

In the analysis, the raw data matrix generated by EST-SSR markers was examined using the GenAlEx 6.5102 project (Peakall and Smouse, 2012). This encompassed parameters such as Shannon’s information index (*I*), observed number of alleles (*N*a), and the number of effective alleles (*N*e). The genetic structure of *E. nutans* populations was investigated via the Bayesian clustering method in Structure v.2.3.4 (Falush et al., 2007). Determination of the optimal number of clusters (K) involved 20 independent runs for each *K* value ranging from 1 to 18. Structure Harvester v0.6.94 (Earl and Vonholdt, 2012) facilitated this analysis, each run comprising 500,000 Monte Carlo Markov Chain (MCMC) replicates. An admixture model was utilized, incorporating a burn-in period of 10,000 replicates. The Δ*K* method (Evanno et al., 2005) was employed to estimate the most likely number of clusters. Subsequently, the 20 replicates underwent clustering and permutation using CLUMPP v1.1 with the LargeK Greedy algorithm (Jakobsson and Rosenberg, 2007). To explore genetic differentiation at the population level, an analysis of molecular variance (AMOVA) was conducted within the GenAlEx 6.5102 project. Additionally, Barrier v2.2, based on Monmonier’s maximum difference algorithm (Manni et al., 2004), was employed to predict major genetic barriers’ geographical locations between populations.

### 2.5 Genetic differentiation and polymorphism with climatic variables

In investigating the relationships between geographical and environmental factors and population genetic differentiation, population genetic distance indices for SSRs and cpDNA were computed using GenAlEx 6.5102 and DnaSP v5.0, respectively. Pairwise geographical distances were calculated utilizing the “geosphere” package in R, while environmental Euclidean distances were derived from nineteen bioclimatic variables obtained from the WorldClim website (https://www.worldclim.org/). Regression analyses were employed to evaluate isolation by distance (IBD) and isolation by environment (IBE) through the plotting of genetic distance against geographical and environmental Euclidean distances, respectively. These analyses facilitated an exploration of potential influences of geographical and environmental factors on population genetic differentiation.

To delve further into potential local adaptation due to environmental fluctuations, the correlation between climate variables and molecular markers was examined. Considering the high ploidy of *E. nutans*, variations in the detected allele numbers among different molecular markers across populations were anticipated. Assessing adaptive trends at specific sites involved establishing a linear regression relationship between the number of effective alleles and climate variables. This analysis offered insights into potential adaptive alterations in response to environmental factors at the identified loci.

## 3 Results

### 3.1 Haplotype distribution and phylogenetic relationship

The total length of the four cpDNA sequences was 4689 bp, and the lengths of the *trn*H-*psb*A, *trn*L-*trn*F, *mat*K, and *rbc*L regions were 667, 1052, 1538, and 1432 bp, respectively. All sequences were deposited in GenBank under accession numbers: OR421574-OR423017. As cpDNA regions are uniparentally inherited markers, we used these four chloroplast fragments in our subsequent population genetics analysis. Among the 361 individuals sampled from 35 populations of *E. nutans*, a total of 20 variable sites were detected in the combined cpDNA. Genetic diversity indicators for various regions of *E. nutans* can be found in **Table 1**. The DQ4 population exhibited the highest values for number of haplotypes (*h* = 6) and haplotype diversity (*H*d = 0.911), followed by population DQ3 (*h* = 5, *H*d = 0.833). The lowest values were observed in populations RQ1, JD2, and SN1, with a single haplotype each (*h* = 1, *H*d = 0). On the other hand, the GG4 population showed the highest nucleotide diversity (*π* = 1.74 ×10^-3^), followed by population DX2 (*π* = 1.06 ×10^-3^), these comprehensive information regarding sample locations, sample sizes, and descriptive statistics of genetic variation for the populations is available in **Supplementary Table 1**. Analyses of molecular variance (AMOVAs) based on cpDNA sequence data revealed that the variation among populations of *E. nutans* was equivalent to the variation within populations, accounting for 49.76% and 50.24%, respectively (**Table 2**).

**Table 1:** Genetic diversity for four regions of *Elymus nutans*.

Region	cpDNA			Microsatellites			
	*h*	*H*d	*π* (10^-3^)	*N*a	*N*e	*I*	*H*e
Qamdo	15	0.827	0.63	2.52	2.17	0.44	0.28
Nyingchi	6	0.821	0.34	2.87	2.24	0.51	0.33
Lhasa	7	0.742	0.69	3.26	2.49	0.57	0.35
Nagqu	7	0.615	0.28	2.62	2.18	0.42	0.27
Total	19	0.805	0.67	2.65	2.21	0.46	0.32

**Table 2:** AMOVA analysis based on the cpDNA and microsatellites for *E. nutans* populations.

Source of variation	*df*	cpDNA				Microsatellites			
		Sum of squares	Variance component	Total variance	*p*	Sum of squares	Variance component	Total variance	*p*
Among populations	34	1934.22	4.89	49.76%	<0.001	985.46	1.31	38%	<0.001
Within populations	326	1610.56	4.94	50.24%	<0.001	1433.16	2.08	62%	<0.001
Total	360	3544.78	9.83	100%		2418.62	3.39	100%	

**Figure 1:**
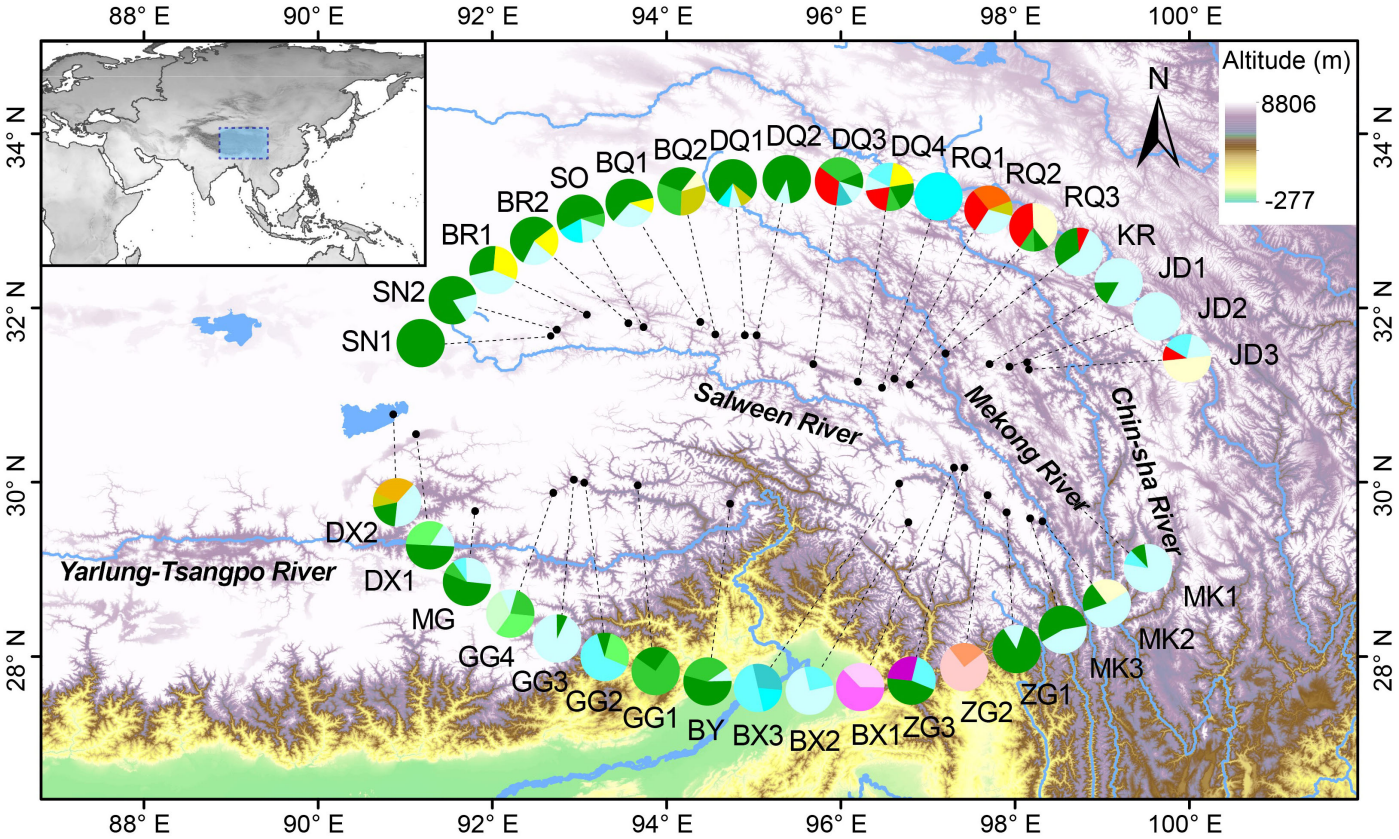
Distribution of 35 *E. nutans* populations sampled in the QTP and 19 chloroplast DNA (cpDNA) haplotypes (H1-H19) screened in this species.

**Figure 2:**
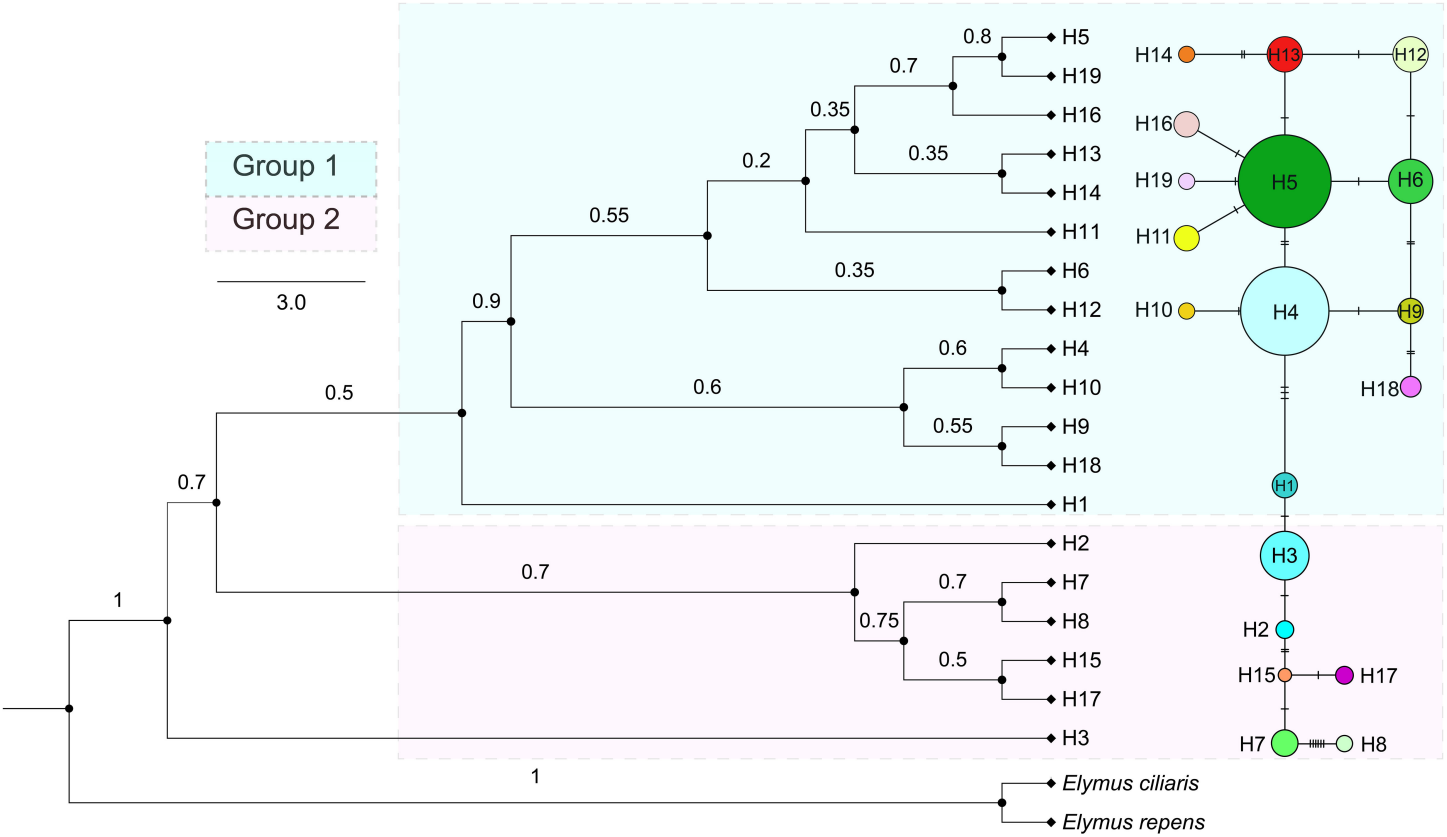
Phylogenetic tree and haplotype structure of *E. nutans* based on cpDNA sequences. Only bootstrap values higher than 0.5 are denoted above branches, the color combination of each circle represents the composition of different haplotypes (H1 – H19), and the size of the circle is proportional to the population size.

**Figure 3:**
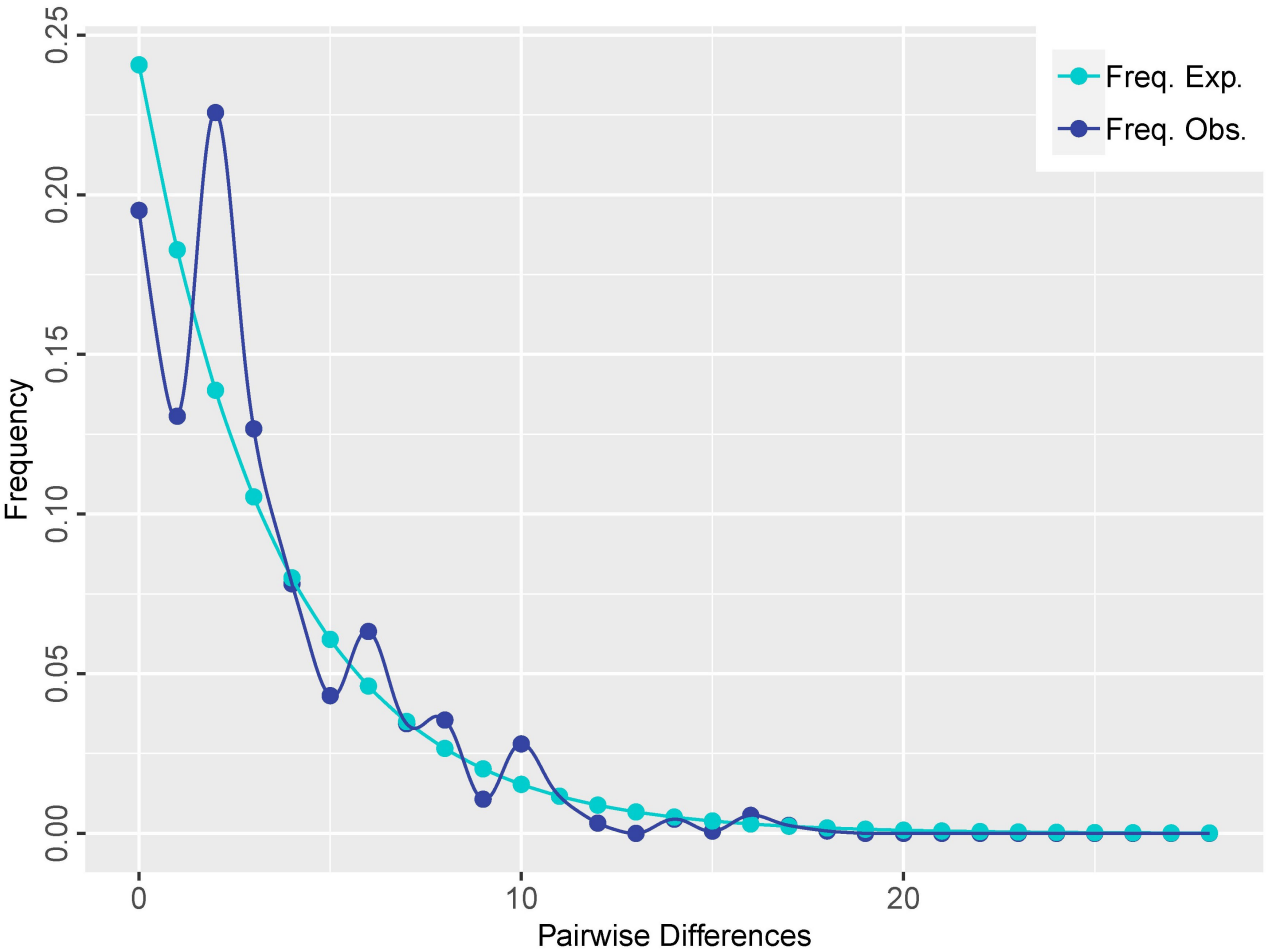
Mismatch distribution analysis plots based on cpDNA sequences for *E. nutans* populations.

A total of 19 distinct haplotypes (H1–H19) were identified, and the spatial distribution of each population, along with its corresponding haplotype composition, is illustrated in **Figure 1**. The differentiation among haplotype categories is visually discernible through color distinctions, as demonstrated in the haplotype network depicted in **Figure 2**. Both the ML tree and the haplotype network consistently exhibit correspondence in **Figure 2**. Based on the distribution patterns in the ML tree and haplotype network, the haplotypes can be systematically grouped into two categories. Group1 comprises 13 haplotypes, prominently featuring the most widely distributed and frequently occurring haplotype H4, alongside its subsequent haplotype H5. These two extensively distributed haplotypes hold central positions within the network diagram, suggesting their potential status as ancestral haplotypes. Additionally, Group1 encompasses 7 unique haplotypes, with H13, H14, H16, and H18 exclusive to Qamdo, while H11 exclusively appears in Nagqu. In contrast, Group2 consists of 6 haplotypes, none of which are observed in Nagqu. Notably, Group2 includes 3 haplotypes from Qamdo (H2, H15, H17) and 1 haplotype H8 from Nyingchi. To gain insight into the historical dynamics of *E. nutans*, mismatch distribution analysis revealed a multimodal pattern (**Figure 3**), and neutrality tests showed that both Fu and Li’s F* (Fu and Li’s F* = 1.22427, *p* > 0.10) and Tajima’s D value (Tajima’s D = –0.07339, *p*>0.10) were not statistically significant. These results do not support population expansions in *E. nutans*.

### 3.2 Genetic diversity, population structure and genetic barriers analysis

Nine EST-SSR markers used to assess the diversity of *E. nutans* populations exhibited polymorphism (**Supplementary Table 2**). The observed allele number (*N*a) detected by each primer ranged from 1.37 (EN91) to 3.57 (EN62), with an average of 2.65. The effective number of alleles (*N*e) per locus ranged from 1.14 (EN91) to 3.30 (EN62), with a mean of 2.21. The average Shannon’s information index (*I*) was 0.46, varying from 0.15 (EN91) to 0.74 (EN5). The observed heterozygosity (*H*o) and expected heterozygosity (*H*e) varied across primers, with the lowest values observed in EN91 (0.06 and 0.11, respectively) and the highest in EN67 (0.99 and 0.55, respectively). The overall average *H*o and *H*e were 0.32 and 0.29, respectively. **Table 1** provides an overview of the genetic variability at the population level. The *N*a and *N*e per population ranged from 1.56 (MK2) to 3.56 (MG) and from 1.55 (MK2) to 2.82 (ZG3), with mean values of 2.65 and 2.21, respectively. Shannon’s information index (*I*) and expected heterozygosity (*H*e) detected in each population ranged from MK2 (*I* = 0.12; *H*e = 0.08) to ZG3 (*I* = 0.74; *H*e = 0.46), with averages of 0.46 and 0.32, respectively.

**Figure 4:**
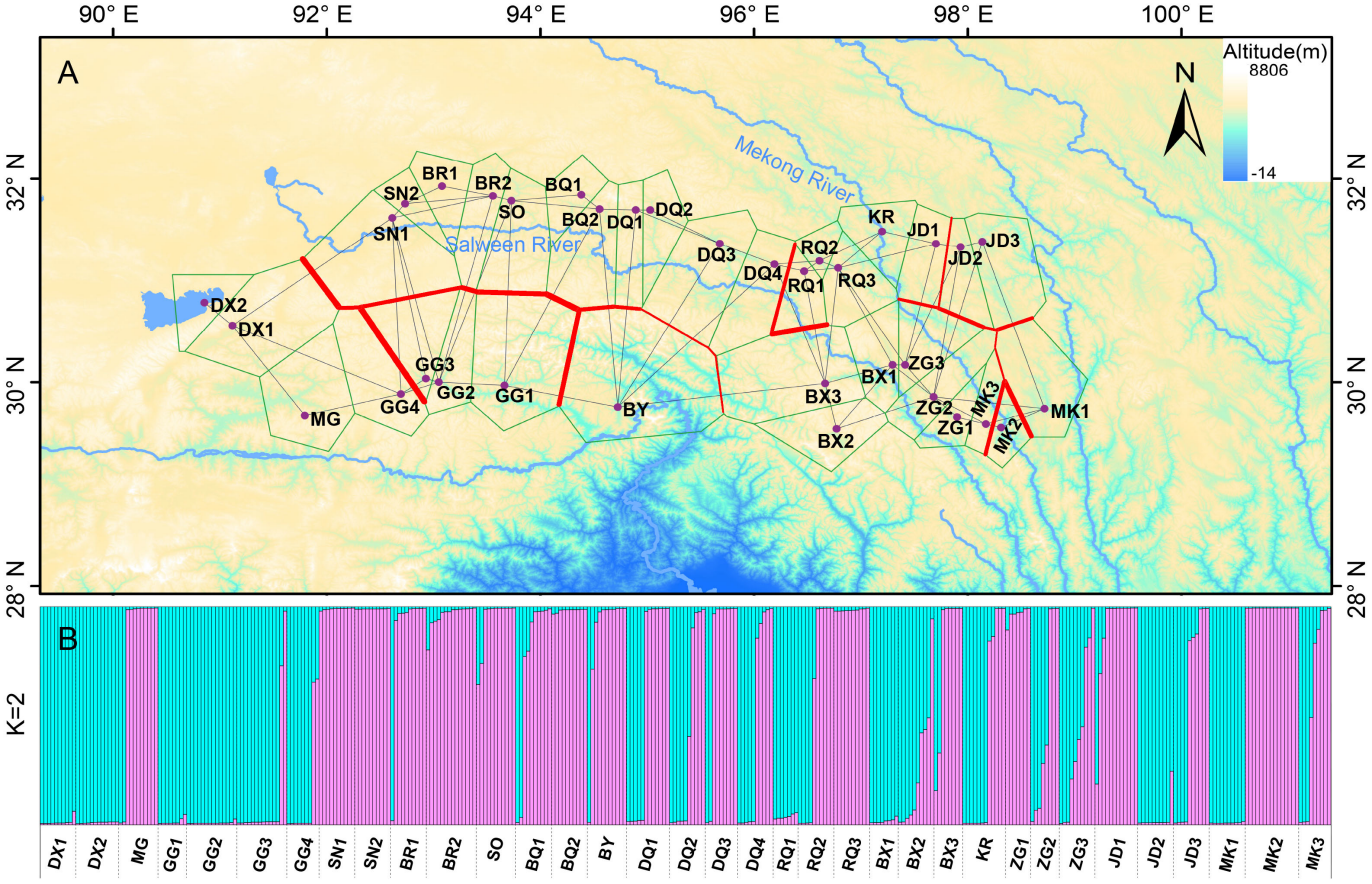
Results of Barrier and Structure analysis among the *E. nutans* populations based on EST-SSRs. **(A)** Bold red lines in the map represented the genetic barrier revealed by Barrier. **(B)** Clustering patterns of the 361 individuals from the 35 *E. nutans* populations by Structure when K = 2, and each individual represented by a vertical-colored line.

The software Barrier was employed to identify genetic barriers among the 35 populations, represented by red lines in **Figure 4A**. The study confirmed that the Salween River serves as a significant genetic barrier in the region. Additionally, crisscrossing genetic barriers were observed near the Mekong River in the eastern part of the study area. The Bayesian cluster analysis (Structure) based on microsatellite data indicated the presence of three optimal clusters (**Supplementary Figure S1**), suggesting that the 361 individuals likely belong to two main genetic clusters. Using CLUMPP to determine the most optimal of the 20 replicates, a plot of the structure of the 35 populations was constructed (**Figure 4B**). Despite a considerable degree of hybridization between populations, their genetic backgrounds can still be differentiated through genetic barriers. Furthermore, the AMOVA results demonstrated that a significant proportion (62%) of the genetic variation exists within the 35 *E. nutans* populations, while 38% of the genetic variation is found among populations (**Table 2**).

### 3.3 Effects of ecogeographical factors on genetic divergence and adaptation

**Figure 5:**
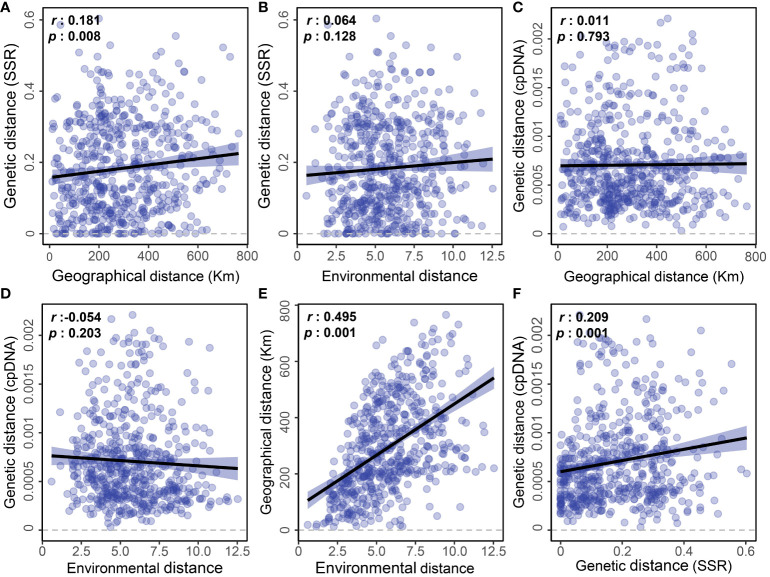
Linear regressions showing pairwise geographic, environmental, and genetic distances detected by EST-SSR and cpDNA sequencing among the *E. nutans* populations. **(A)** Genetic distance (SSR) and Geographical distance; **(B)** Genetic distance (SSR) and Environmental distance; **(C)** Genetic distance (cpDNA) and Geographical distance; **(D)** Genetic distance (cpDNA) and Environmental distance; **(E)** Geographical distance and Environmental distance; **(F)** Genetic distance (cpDNA) and Genetic distance (SSR).

Based on the Mantel test using pairwise distance values, our study revealed that geographic distance had a weak effect on the genetic differentiation detected by microsatellites (**Figure 5A**, *r*=0.181; *p*=0.008), while environmental distance did not show a significant impact (**Figure 5B**, *r*=0.064; *p*=0.128). However, there was no significant correlation between either geographic distance or environmental distance and the population genetic differentiation detected by cpDNA (**Figures 5C, D**). Nevertheless, we did observe a significant correlation between geographic distance and environmental distance among sampling points (**Figure 5E**, *r* = 0.495; *p* = 0.001), as well as a significant correlation between genetic distances among populations detected by cpDNA and microsatellites (**Figure 5F**, *r* = 0.209; *p* = 0.001).

**Figure 6:**
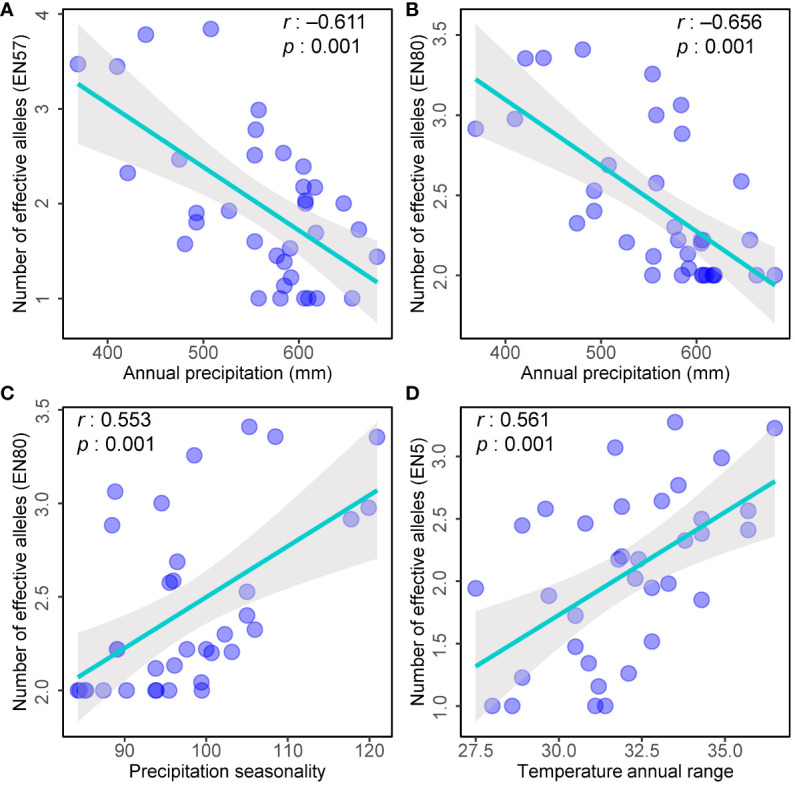
Scatter plots showing the relationships between the environmental factors and number of effective alleles (Ne) in each population. **(A)** Number of effective alleles (EN57) and Annual precipitation; **(B)** Number of effective alleles (EN80) and Annual precipitation; **(C)** Number of effective alleles (EN80) and Precipitation seasonality; **(D)** Number of effective alleles (EN5) and Temperature annual range.

The variation of alleles is likely attributed to adaptation to external environmental factors (Ramírez-Valiente et al., 2010). Here, we examined the correlation between the number of effective alleles (*N*e) in each population and the environmental factors. Three of the markers detected a significant correlation between the number of effective alleles detected and ecogeographical data (**Figure 6**). Specifically, the *N*e detected by marker EN57 and EN80 exhibited a negative correlation with annual precipitation (**Figures 6A, B**), while the *N*e detected by marker EN80 showed a significant positive correlation with the seasonal variability of precipitation (**Figure 6C**). Additionally, the *N*e detected by marker EN5 displayed a significant positive correlation with the annual temperature range (**Figure 6D**). Upon annotating these loci with gene functions within the database, it was revealed that the marker EN57 locus is linked to glutathione S-transferase T1. In contrast, the marker EN5 and EN80 loci exhibited homology with hypothetical proteins in species such as *Aegilops tauschii*, *Hordeum vulgare*, and *Setaria italica*, among others. Notably, the precise functions of these hypothetical proteins remain uncharacterized and warrant further accurate annotation.

## 4 Discussion

### 4.1 Population genetic variation

Genetic diversity, encompassing the total genetic variation among individuals within different populations of a species, serves as a crucial indicator of population adaptability to changing environments (Bell and Collins, 2008). Given the climate sensitivity of the QTP region, investigating the genetic diversity of species in this area holds significant importance (Wambulwa et al., 2021). *E. nutans*, a prominent herbaceous plant widely distributed in the QTP, has demonstrated exceptionally high levels of genetic variation through various approaches, including phenotype, ISSR, and SSR analyses (Chen et al., 2009; Li et al., 2023). However, prior studies on genetic variation primarily relied on individual or pooled genotyping resources, which might not sufficiently capture the spatial variation of genetic diversity among populations within this species.

To bridge the existing knowledge gap, a comprehensive analysis was undertaken, examining genetic variability among 361 specimens drawn from 35 distinct populations throughout the QTP region, with a specific focus on variations in nuclear and plastid fragments. The study unveiled substantial genetic diversity in both nuclear and plastid genes. Notably, the number of alleles (*N*a = 2.65) and effective alleles (*N*e = 2.21) identified through EST-SSR markers were in close alignment with those reported in a prior investigation of the closely associated and sympatric species, *E. breviaristatus* (Li et al., 2022; *N*a = 2.71; *N*e = 2.28). Nevertheless, it is imperative to acknowledge that the Shannon information index (*I*) recorded for the *E. nutans* population (*I* = 0.46) was significantly elevated in comparison to that of *E. breviaristatus* (*I* = 0.13), underscoring a distinctive and intriguing pattern of genetic diversity. As a species with an extensive distribution, *E. nutans* demonstrates a more homogeneous allele distribution, potentially attributed to its propensity to sustain larger population sizes and engage in more substantial gene flow across various populations (Magota et al., 2021). Conversely, the endangered status of *E. breviaristatus* may exert limitations on gene flow and genetic exchange, culminating in a diminished Shannon’s information index (*I*). This stark contrast elucidates the impact of species distribution and conservation status on genetic diversity and gene flow within these species.

A high *H*d (0.805) and *π* (0.67×10^-3^) were revealed in our study, indicating values lower than those observed in the related and widespread species of *Elymus sibiricus* in the QTP region detected based on cpDNA (*H*d = 0.834, *π* = 1.08×10^-3^). Conversely, these values surpassed those documented for *E. sibiricus* populations in Xinjiang and northern China, which exhibit respective *H*d values of 0.527 and 0.543, and π values of 0.042×10^-3^ and 0.53×10^-3^, respectively (Xiong et al., 2022). Similarly, this high degree of genetic diversity has also been confirmed in other plants on the Tibetan Plateau, such as *Stellera chamaejasme* (*H*d = 0.834) (Zhang et al., 2010), *Iris lozyi* (*H*d = 0.820) (Zhang et al., 2021). This observation corroborates the notion that the QTP serves as a focal point for the differentiation in most species, including *Elymus* species. Further, on a population scale, specific groups displaying elevated haplotype counts and notable haplotype diversity, as exemplified by populations such as DQ3, DQ4, RQ2, and others, are strategically positioned within the geographical span delimited by the Salween River and the Mekong River. These observations substantiate the hypothesis that this geographic region holds substantial significance as a sanctuary during ice ages for *E. nutans*. Furthermore, the locale has historically held a pivotal role as a biogeographic demarcation within the East Asian floral context, commonly recognized as the Ward Line-Mekong-Salween Divide (MSD) (Ward, 1921; Luo et al., 2017), which is highly suggestive of its potential as a hub for *E. nutans* diversification. In addition, this hypothesis is supported by the previous findings of Yu et al (Yu et al., 2019), who identified this field as one of the nine evolutionary hotspots in the QTP, bolsters the proposition of its paramount significance in the evolutionary trajectory of *E. nutans*, namely the eastern part of Nyenchen Tanglha Mountains.

### 4.2 Genetic structure and barriers

The analysis of cpDNA haplotypes and microsatellite structures in this study suggests that the genetic differentiation among the 35 populations of *E. nutans* lacks distinct definition. This conclusion is supported by the presence of individuals displaying mixed genetic backgrounds within the majority of populations. Moreover, the AMOVA results emphasize that genetic variation primarily arises within populations, with comparatively lower levels of genetic divergence observed among populations. We attribute this observed divergence to *E. nutans*’ wider ecological niche and larger effective population size, which likely enhance individuals’ migration and dispersion across diverse habitats, facilitating gene flow among populations. Such occurrences are common among widely distributed plant species and are pivotal in maintaining genetic diversity and adaptability within populations (Broadhurst et al., 2018; Kahl et al., 2021; Veto et al., 2023).

Nonetheless, there still exist pronounced genetic barriers among certain adjacent populations. These barriers could be attributed to specific factors such as geographic isolation, environmental differences, or ecological niche divergence (Ryan et al., 2017; Yu et al., 2021), which collectively restrict gene flow among populations. As indicated by the results obtained from the Barrier analysis (**Figure 3**), our study reveals that the genetic barriers detected are primarily aligned with the Salween River and the Mekong River, impeding gene flow between populations situated on opposite sides of these rivers. The Ward Line-MSD, formed by these two rivers, holds significance as a major biogeographic boundary within the East Asian plant region (Ward, 1921). It has been extensively studied and confirmed to exist in various species, such as *Marmoritis complanatum*, *Koenigia forrestii*, and *Sinopodophyllum hexandrum* (Li et al., 2011; Luo et al., 2017; Rana et al., 2023). Our research, focusing on the intraspecific genetic differentiation of populations and operating at a finer scale, further underscores the importance of MSD in the dynamic process of species differentiation. The elucidation of these intricacies significantly enriches our comprehension of species evolution and ecological dynamics. Such an enhanced understanding, in turn, facilitates the implementation of more refined and effective strategies for the collection, conservation, and management of the germplasm resources pertaining to this species.

### 4.3 The role of ecogeographical factors on genetic divergence and adaptation

Although our study highlights the extensive effect of the MSD concerning the genetic separation of *E. nutans* populations, which restricts gene flow and leads to prompt genetic divergence, a noteworthy phenomenon presents itself in the form of a weak yet statistically significant pattern of isolation by distance (IBD) among populations only demonstrated via microsatellite data. In contrast to the robust IBD pattern uncovered in our earlier study of *E. breviaristatus* populations (Li et al., 2022), the relatively weaker or even absent IBD pattern discerned in *E. nutans* populations further reinforces the notion of heightened dispersal capabilities within these populations. However, the insights gleaned from the cpDNA analysis do not corroborate this phenomenon. It is posited that this disparity may originate from the fundamental differences in transmission modes, rates of genetic drift, and migration patterns inherent to nuclear and plastid DNA, culminating in the divergent genetic configurations manifested in these two distinct genetic substrates (Soltis and Kuzoff, 1995).

Perennial plants strategically accumulate diverse allelic variants in response to various environmental conditions, reflecting their adaptive mechanism to external selection pressures (Castillo et al., 2010; Sork et al., 2010; Raschke et al., 2015). In our investigation, we identified significant divergence within three analyzed loci among populations situated in regions marked by more extreme climatic conditions, such as low precipitation or imbalances in precipitation and temperature. Of particular significance, notable correlations were observed between the number of effective alleles and specific environmental factors, indicating a discernible influence of natural selection on these genetic markers, thereby facilitating localized genetic differentiation. While two of these loci lack prior annotations, it is noteworthy that marker EN67 is unequivocally associated with a glutathione S-transferase T1 in wheat and *Arabidopsis*. Glutathione S-transferase, an essential component of the glutathione antioxidant system, plays a pivotal role in managing oxidative stress and detoxifying harmful compounds within plants (Sappl et al., 2009; Nianiou-Obeidat et al., 2017). We posit that this functional attribute may contribute to allelic divergence observed in arid regions. Additionally, although the marker EN5 and EN80 motifs lack established annotations, we hypothesize that they are linked to mechanisms enabling adaptation to climatic extremes. These motifs hold promise as potential candidate genes for genetic breeding in wheat plants, particularly to enhance resilience against environmental challenges. Further research is warranted to unravel the precise functional implications of these loci and their roles in plant adaptation.

Collectively, our findings, coupled with our prior results, implies that concerning gene loci affected by environmental selection, the lack of a clear-cut isolation by environment (IBE) pattern signifies a more intricate association between genetic differentiation and environmental adaptation than previously presumed. This complexity likely arises from a confluence of interacting factors, highlighting the multifaceted nature of the evolutionary processes governing population differentiation and adaptation.

## 5 Conclusions

In this comprehensive study, the genetic dynamics of *E. nutans* populations across the QTP were explored using a combination of cpDNA and microsatellite analyses. Significant genetic diversity within and among populations was revealed through the analysis of haplotype distribution and phylogenetic relationships. Mismatch distribution analysis and neutrality tests indicated a complex demographic history with no evidence of recent population expansion. The examination of population genetic differentiation unveiled the significant role of geographic barriers in shaping the genetic landscape of *E. nutans*, with the Salween River and Mekong River identified as potent genetic boundaries that impede gene flow between populations. Although the impact of geographic distance on genetic differentiation appears to be minimal, significant correlations have been identified between certain microsatellite loci and environmental factors, suggesting potential adaptability of these loci to climatic challenges. In summary, this study unveils the intricate genetic pathways of *E. nutans* within the dynamic QTP. The findings underscore the directive influence of geographical barriers and ecological factors on genetic differentiation and adaptation. The insights garnered from this research hold substantial importance for the conservation of germplasm resources and resistance breeding in the context of an ever-changing environment.

## Data availability statement

The datasets presented in this study can be found in online repositories. The names of the repository/repositories and accession number(s) can be found below: https://www.ncbi.nlm.nih.gov/genbank/, OR421574–OR423017.

## Author contributions

JL: Conceptualization, Investigation, Methodology, Writing – original draft. XL: Investigation, Methodology, Writing – original draft. CZ: Methodology, Resources, Validation, Writing – original draft. QZ: Conceptualization, Validation, Writing – review & editing. SC: Conceptualization, Funding acquisition, Resources, Supervision, Writing – review & editing.
