# Supplementary material for: Phylogeographic analysis reveals extensive genetic variation of native grass Elymus nutans (Poaceae) on the Qinghai-Tibetan plateau
Source: Front Plant Sci. 2024 Mar 11;15:1349641. doi: 10.3389/fpls.2024.1349641 (PMC10961384; doi:10.3389/fpls.2024.1349641)
Supplement: Supplementary file 1 [file DataSheet_1.docx]

Supplementary Material

## Supplementary Figures


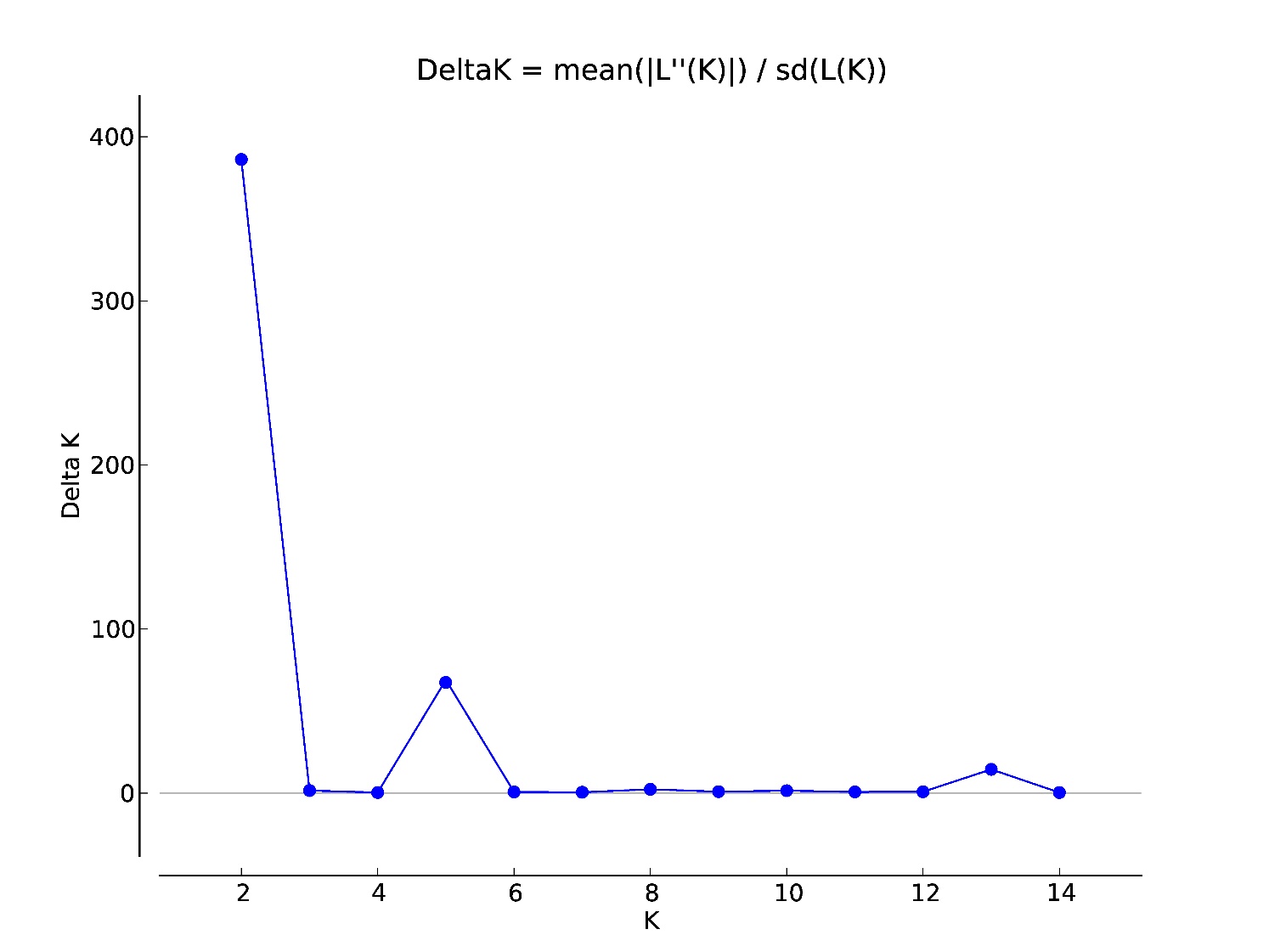


**Supplementary Figure 1.** *K* model with an elevated Δ*K* values calculated for *K* varying from 2 to 14.

**Supplementary Table 1.** Details of sample locations, sample sizes and descriptive statistics of genetic variability for 35 populations of *Elymus nutans.*

| Region | District | Pop Code | Pop Size | Latitude (°E) | Longitude (°N) | Altitude (m) | cpDNA | | | Microsatellites | | | |
| --- | --- | --- | --- | --- | --- | --- | --- | --- | --- | --- | --- | --- | --- |
|  |  |  |  |  |  |  | *h* | *H*d | *π*(10^-3^) | *N*a | *N*e | *I* | *H*e |
| Qamdo | Markam | MK1 | 10 | 29.74 | 98.72 | 3530 | 3 | 0.378 | 0.26 | 2.22 | 1.94 | 0.36 | 0.24 |
|  | Markam | MK2 | 15 | 29.55 | 98.32 | 3920 | 3 | 0.648 | 0.20 | 1.56 | 1.55 | 0.12 | 0.08 |
|  | Markam | MK3 | 9 | 29.59 | 98.17 | 3966 | 2 | 0.556 | 0.24 | 3.00 | 2.62 | 0.57 | 0.37 |
|  | Zogang | ZG1 | 7 | 29.65 | 97.90 | 3902 | 2 | 0.286 | 0.12 | 2.44 | 2.13 | 0.42 | 0.27 |
|  | Zogang | ZG2 | 8 | 29.85 | 97.68 | 3909 | 2 | 0.429 | 0.73 | 2.89 | 2.48 | 0.60 | 0.39 |
|  | Zogang | ZG3 | 10 | 30.17 | 97.42 | 4110 | 3 | 0.600 | 0.91 | 3.44 | 2.82 | 0.74 | 0.46 |
|  | Baxoi | BX1 | 8 | 30.17 | 97.30 | 4593 | 2 | 0.536 | 0.46 | 2.11 | 1.88 | 0.32 | 0.22 |
|  | Baxoi | BX2 | 10 | 29.98 | 96.67 | 3776 | 3 | 0.689 | 0.17 | 2.22 | 2.02 | 0.41 | 0.28 |
|  | Baxoi | BX3 | 8 | 29.54 | 96.78 | 4118 | 2 | 0.429 | 0.46 | 2.67 | 2.31 | 0.48 | 0.32 |
|  | Dêngqên | DQ1 | 12 | 31.69 | 94.90 | 4525 | 4 | 0.455 | 0.32 | 2.67 | 2.24 | 0.50 | 0.32 |
|  | Dêngqên | DQ2 | 10 | 31.69 | 95.03 | 3827 | 2 | 0.200 | 0.09 | 2.56 | 2.10 | 0.48 | 0.31 |
|  | Dêngqên | DQ3 | 10 | 31.36 | 95.68 | 3738 | 5 | 0.833 | 0.52 | 2.78 | 2.30 | 0.45 | 0.30 |
|  | Dêngqên | DQ4 | 10 | 31.16 | 96.20 | 4108 | 6 | 0.911 | 0.71 | 3.33 | 2.50 | 0.65 | 0.40 |
|  | Riwoqê | RQ1 | 7 | 31.09 | 96.47 | 4560 | 1 | 0.000 | 0.00 | 1.78 | 1.70 | 0.25 | 0.18 |
|  | Riwoqê | RQ2 | 10 | 31.19 | 96.62 | 3786 | 4 | 0.800 | 0.58 | 2.78 | 2.24 | 0.50 | 0.31 |
|  | Riwoqê | RQ3 | 10 | 31.12 | 96.79 | 3708 | 4 | 0.733 | 0.19 | 2.11 | 1.88 | 0.28 | 0.18 |
|  | Karuo | KR | 12 | 31.48 | 97.20 | 3340 | 3 | 0.591 | 0.26 | 2.78 | 2.37 | 0.50 | 0.31 |
|  | Jomda | JD1 | 12 | 31.36 | 97.71 | 4506 | 2 | 0.303 | 0.13 | 2.11 | 1.93 | 0.33 | 0.22 |
|  | Jomda | JD2 | 10 | 31.32 | 97.94 | 3952 | 1 | 0.000 | 0.00 | 1.67 | 1.58 | 0.20 | 0.14 |
|  | Jomda | JD3 | 10 | 31.38 | 98.14 | 3736 | 4 | 0.733 | 0.76 | 3.22 | 2.72 | 0.61 | 0.37 |
|  | Total | | | | | | 15 | 0.827 | 0.63 | 2.52 | 2.17 | 0.44 | 0.28 |
| Nyingchi | Bayi | BY | 10 | 29.75 | 94.73 | 3350 | 3 | 0.618 | 0.19 | 2.89 | 2.34 | 0.50 | 0.33 |
|  | Gongbogyamda | GG1 | 8 | 29.96 | 93.67 | 3294 | 2 | 0.429 | 0.09 | 3.00 | 2.34 | 0.54 | 0.33 |
|  | Gongbogyamda | GG2 | 14 | 30.00 | 93.06 | 3582 | 3 | 0.604 | 0.63 | 3.00 | 2.22 | 0.56 | 0.37 |
|  | Gongbogyamda | GG3 | 14 | 30.03 | 92.93 | 3646 | 2 | 0.143 | 0.06 | 2.89 | 2.01 | 0.47 | 0.28 |
|  | Gongbogyamda | GG4 | 9 | 29.88 | 92.70 | 3969 | 4 | 0.806 | 1.74 | 2.56 | 2.29 | 0.49 | 0.32 |
|  | Total | | | | | | 6 | 0.821 | 0.34 | 2.87 | 2.24 | 0.51 | 0.33 |
| Lhasa | Maizhokunggar | MG | 11 | 29.67 | 91.80 | 4236 | 4 | 0.673 | 0.41 | 3.56 | 2.67 | 0.63 | 0.39 |
|  | Damxung | DX1 | 10 | 30.78 | 90.86 | 4840 | 4 | 0.778 | 0.29 | 3.11 | 2.55 | 0.53 | 0.32 |
|  | Damxung | DX2 | 12 | 30.55 | 91.12 | 4325 | 3 | 0.667 | 1.06 | 3.11 | 2.26 | 0.54 | 0.33 |
|  | Total | | | | | | 7 | 0.742 | 0.69 | 3.26 | 2.49 | 0.57 | 0.35 |
| Nagqu | Seni | SN1 | 10 | 31.75 | 92.74 | 4254 | 1 | 0.000 | 0.00 | 3.33 | 2.48 | 0.57 | 0.35 |
|  | Seni | SN2 | 10 | 31.75 | 92.74 | 4254 | 2 | 0.356 | 0.15 | 1.89 | 1.82 | 0.26 | 0.18 |
|  | Biru | BR1 | 10 | 31.92 | 93.09 | 4434 | 3 | 0.733 | 0.33 | 2.89 | 2.35 | 0.45 | 0.28 |
|  | Biru | BR2 | 14 | 31.83 | 93.56 | 4369 | 3 | 0.626 | 0.23 | 2.67 | 2.29 | 0.48 | 0.31 |
|  | Sog | SO | 11 | 31.78 | 93.74 | 3950 | 4 | 0.691 | 0.46 | 2.33 | 1.91 | 0.35 | 0.21 |
|  | Baqên | BQ1 | 10 | 31.84 | 94.39 | 4127 | 3 | 0.600 | 0.24 | 2.89 | 2.41 | 0.53 | 0.33 |
|  | Baqên | BQ2 | 10 | 31.70 | 94.56 | 4154 | 4 | 0.800 | 0.34 | 2.33 | 1.97 | 0.32 | 0.20 |
|  | Total | | | | | | 7 | 0.615 | 0.28 | 2.62 | 2.18 | 0.42 | 0.27 |
| All populations | | | | | | | 19 | 0.805 | 0.67 | 2.65 | 2.21 | 0.46 | 0.32 |

**Supplementary Table 2.** Characterization of the EST-SSRs primer pairs in *E. nutans*.

| Primer | Forward primer (5'-3') | Reverse primer (5'-3') | *N*a | *N*e | *I* | *H*o | *H*e |
| --- | --- | --- | --- | --- | --- | --- | --- |
| EN5 | GCTAGGAGCCAGAATTAAGGGA | GGGTCAGTGTCTCGGTTTGT | 2.89 | 2.05 | 0.74 | 0.24 | 0.43 |
| EN57 | CTCGGACTGGACTTCTCAGC | GGGAAAACGGTGGCGAGATA | 2.74 | 1.95 | 0.68 | 0.21 | 0.39 |
| EN62 | ACTTTCTTGATGGACGGGGG | ATCATCACAGCGACGGTGAG | 3.57 | 3.30 | 0.39 | 0.50 | 0.28 |
| EN67 | GCCCCTCCCCCATTTGTTG | CAAGGTCTGCATCCATTGGC | 2.57 | 2.24 | 0.84 | 0.99 | 0.55 |
| EN80 | CGCGAGCATTTTCGTAGCAG | GTCAAAGTTGGGAGGGGGAG | 2.97 | 2.43 | 0.20 | 0.13 | 0.12 |
| EN83 | CCTCCTCCCTCCCGTAGTTC | CGAAAATGCCAGGTTTCCGG | 2.77 | 2.53 | 0.33 | 0.36 | 0.23 |
| EN91 | ACAAGGTTGGGAACGCAGAA | CAGCAGCTCACCATGGTCA | 1.37 | 1.14 | 0.15 | 0.06 | 0.11 |
| EN98 | AGCTTGGTGATTCCGCTTGA | AACAGCCGATATAGCCTGGC | 1.80 | 1.39 | 0.38 | 0.08 | 0.24 |
| EN99 | CTGTTCGCCTCAGCTGAAGA | AGCACTAGTTACCCGCAACC | 3.17 | 2.83 | 0.41 | 0.34 | 0.28 |
| Average | | | 2.65 | 2.21 | 0.46 | 0.32 | 0.29 |
